# Woody seeds and seedlings are unresponsive to herbivore kairomones

**DOI:** 10.1093/aobpla/plag013

**Published:** 2026-02-26

**Authors:** Kevin C Headrick, Brooke A Pellegrini, Eirette M Santiago, Katherine M Overstrum, Santhi P Bhavanam, Colin M Orians, John L Orrock, Evan L Preisser

**Affiliations:** Department of Biology, Tufts University, 200 College Ave, Medford, MA 02155, United States; Department of Biological Sciences, University of Rhode Island, 120 Flagg Rd, Kingston, RI 02881, United States; Department of Biological Sciences, University of Rhode Island, 120 Flagg Rd, Kingston, RI 02881, United States; Department of Biological Sciences, University of Rhode Island, 120 Flagg Rd, Kingston, RI 02881, United States; Department of Biology, Tufts University, 200 College Ave, Medford, MA 02155, United States; Department of Entomology, Plant Pathology, and Weed Science, New Mexico State University, 945 College Dr, Las Cruces, NM 88003, United States; Department of Biology, Tufts University, 200 College Ave, Medford, MA 02155, United States; Department of Integrative Biology, University of Wisconsin, 250 N Mills St, Madison, WI 53706, United States; Department of Biological Sciences, University of Rhode Island, 120 Flagg Rd, Kingston, RI 02881, United States

**Keywords:** herbivory, kairomone, germination, mollusk, risk

## Abstract

Herbivory is particularly threatening to young plants that lack the resources needed to survive an attack. Seeds and seedlings should thus benefit greatly from using pre-attack cues to induce defence before damage. Locomotion mucus from slugs, generalist herbivores that consume young plants, has been shown to speed germination, slow growth, and increase both chemical defences and resistance to herbivores in several herbaceous plants. Whether woody species exhibit similar responses has not been tested. *Arion subfuscus*, an invasive slug in the eastern USA, is a major herbivore of young sugar maples (*Acer saccharum*); we explored the effect of its locomotion mucus on *Ac. saccharum* seeds and seedlings. We exposed sugar maple seeds and seedlings to mucus and measured germination speed and rate, seed susceptibility to slugs, seedling emergence, growth, chemical defences, and foliar susceptibility to *Lymantria dispar* and *Ar. subfuscus*. Contrary to our expectations and previous findings with herbaceous species, we found that mucus had no effect on these performance or resistance traits. Habituation to repeated cue exposure or the limited coevolutionary history between *Ac. saccharum* and *Ar. subfuscus* could be the reason for the lack of an observed response by the plants. Further studies should investigate the effects of kairomones using a short-term cue exposure procedure or by using a woody plant species and native slug herbivore with a coevolutionary history. Understanding how woody plants respond to kairomones would provide insight into the risk and defence strategies used by long-lived species in crucial early life stages.

## Teaser

Our study looked at whether Acer saccharum seeds or seedlings responded to the application of mucus from Arion subfuscus slugs as an indicator of potential future attack. Using validated methods of mucus application that prompted changes in germination and growth in Brassica nigra, we found that mucus application has no effect on sugar maple seeds or seedling response traits. Traits included germination, emergence, shoot height, foliar chemistry, and resistance to herbivory on several tissue types.

## Introduction

Attack by herbivores can have significant effects on plant survival and reproduction (Hendrix 1988, Hawkes and Sullivan 2001, Maron and Crone 2006). However, the production of chemical defences against herbivores may be costly, using resources that could otherwise be directed to growth and/or reproduction if herbivory is unlikely or inconsequential (Orrock et al. 2015). Induced defences provide one means to maximize the benefits of needed defence while minimizing the costs of inducing defences that are not truly needed (Huot et al. 2014, He et al. 2022, Monson et al. 2022). However, the optimal deployment of induced defences requires some estimate of impending risk of herbivory, such that plants often rely upon attack-related cues to induce defence (Karban et al. 1997). These cues include direct physical damage, insect saliva (Felton 2008), and damage-induced plant volatiles (Heil and Karban 2010). Reliance on such high-quality information reflects the fact that plant defence induction, e.g. the activation of the jasmonic acid or salicylic acid pathways, is often energetically costly (Huot et al. 2014). Plants that divert energy to unnecessary defence may not grow as quickly and risk being outcompeted for light, nutrients, and other resources. Although induced defences are widespread in plants (Karban et al. 1997, 1999, Agrawal 1999), they also exhibit significant variation in time and space (Boege and Marquis 2005, Barton and Koricheva 2010). As a result, understanding the factors that are reliably associated with induced defences is a primary goal of plant-herbivore ecology (Agrawal 2011).

While mature plants can survive even intense herbivory (Sheriff et al. 2020), their early life stages are far more vulnerable. The small size of seeds/seedlings and their relative lack of energetic reserves can make it difficult or impossible for them to recover from herbivory, suggesting the importance of detecting and responding to herbivores prior to damage. One means of pre-attack detection involves kairomones, non-attack-related chemical substances emitted by consumers that benefit the potentially consumed species (Schoeppner and Relyea 2009). Kairomone use is common in predator-prey systems, and seeds and seedlings may be under similar selective pressure to avoid or deter attack (Clinchy et al. 2013, Sheriff et al. 2020). Consistent with this hypothesis, both seeds (Zolovs et al. 2022, Pellegrini et al. 2024) and seedlings (Orrock 2013, Falk et al. 2014, Orrock et al. 2018, Overstrum et al. 2026) have been shown to respond to herbivore kairomones, specifically locomotion mucus exuded by molluscan herbivores, by increasing defence (measured as herbivore palatability) at the cost of somatic growth.

While kairomone use has thus far been documented only in herbaceous species, the seeds and seedlings of woody plants are also susceptible to herbivory damage. Seedlings of *Pinus sylvestris,* for example, are most susceptible to herbivory damage within one month of germination, and are primarily damaged by slugs (Nystrand and Granstrom 2000). In addition, a review of ontogenetic differences in the effects of herbivory showed that the seed and seedling stages have lower tolerance for herbivory than sapling and adult stages in both woody and herbaceous seedlings (Massad 2013). Other work on woody species found that seedlings under five cm in height are more likely to suffer herbivore damage (Albrectsen et al. 2004), that seeds subjected to simulated herbivory grew into smaller seedlings (Lorca et al. 2019), and that seeds that experienced herbivory in a natural setting also grew into smaller seedlings (Branco et al. 2002). The seeds and seedlings of woody plants often differ substantially from their herbaceous counterparts, however, in factors such as their structure, chemical and physical defences against herbivory (Chen and Moles 2018, Dalling et al. 2020), capacity to tolerate subsequent herbivory (Armstrong and Westoby 1993), and long germination periods (Chen et al. 2002). These differences make it difficult to predict whether early life stages of woody species are similarly responsive to pre-attack herbivore cues. We report the results of work exploring the responses of seeds and seedlings of a long-lived woody plant (*Acer saccharum*) to the locomotion mucus (kairomone) of an herbivorous slug (*Arion subfuscus*). Both species are foundational in northeastern US forest ecosystems.

*Ac. saccharum*, or sugar maple, is a deciduous tree species with high ecological and economic importance that is best known for its use in the production of maple syrup, a $130 million crop in the United States in 2015 (Lustenhouwer et al. 2012, Matthews and Iverson 2017). Their seeds cold stratify over the winter and after emergence, seedlings grow with opposite leaf pairs (Steingraeber 1982, Nyland 1999). *Ac. saccharum* reaches reproductive maturity by year 50 and reproduces in a masting pattern (Nyland 1999). During a mast year, samaras bound in pairs are released, and seeds are protected by a strong seed coat. Leaves of sugar maples are protected by high concentrations of tannins and other phenolic compounds (Baldwin et al. 1987). As sugar maple leaves mature, they become thicker and tougher, and thus possess physical as well as chemical defences (unpublished data).

*Ar. subfuscus* is a naturalized polyphagous herbivorous slug introduced to North America from Europe in the early 19th century (Chichester and Getz 1973); it is the most abundant slug species in New England (French 2012). *Ar. subfuscus ‘*has the potential for having the greatest impact upon natural communities’ of any northeastern U.S. terrestrial mollusc (Chichester and Getz 1969), and it is frequently found in maple stands (Beyer and Saari 1978). More than half of observed *Acer* genus seedling mortality has been attributed to herbivory from slugs (Pigot and Leather 2008). It is thus likely that *Ar. subfuscus* exerts strong selection pressure on *Ac. saccharum* seeds and seedlings. Also*, Ar. subfuscus* mucus has previously been shown to alter plant growth and defence in *Brassica nigra* (Pellegrini et al. 2024, Overstrum et al. 2026); we exposed *Ac. saccharum* seeds and seedlings to *Ar. subfuscus* locomotion mucus to assess the effect of kairomones on germination, growth, and herbivory.

## Materials and methods

### Study species

Adult *Ar. subfuscus* were collected May–August 2024 from leaf litter, fallen logs, and trees at forested sites near Tufts University and The University of Rhode Island. Slugs were maintained in the lab on a diet of organic produce (romaine lettuce *Lactuca sativa* var. *longifolia*, iceberg lettuce *L. sativa* var. *capitata*, and carrots *Daucus carota*), and leaf litter in 21–24°C terrariums that were regularly misted to maintain humidity.

*Ac. saccharum* seeds were sourced from the same lot from Sheffield’s Seed (Locke, NY, USA). Seeds were kept in a refrigerator at ∼4°C before the experiment. To cold stratify the seeds, seeds were soaked in water for 24 h, placed in petri dishes on moist soil, and then returned to a refrigerator at 4°C for cold stratification. After 30 days, we tested the effects of mucus on seed germination and vulnerability, and later the effects of mucus and methyl jasmonate on seedling responses.

### Seed experiments

#### Cue preparation

Cues were collected, stored, and applied using published methods (Overstrum et al. 2026). Here, the timing of cue application began on day 30 of stratification to synchronize seed exposure with expected slug activity in the spring. Specifically, one day before the start of cue application, 90 mm petri plates (Tufts: *n* = 16, URI: *n* = 20) each received 9.0 g of a 7:2 mixture of water and sifted MiracleGro Potting Mix soil (0.22/0.12/0.17 NPK). Immediately following soil addition, half the petri plates were randomly chosen for the cue treatment; the other half were assigned to the control treatment. Two mature *Ar. subfuscus* (0.75 ± 0.25 g per individual) were added to each of the cue treatment petri plates; control plates did not receive slugs. Following slug addition, all petri plates were covered, interspersed, and stored in a dark cabinet at 22–23°C.

After 24 h, the plates were taken from the cabinet, the slugs removed, and the soil mixed to evenly disperse the deposited mucus. All the petri dishes were frozen at −20°C, then thawed the night before being used. Overstrum et al. (2026) showed that the cue signal remains effective when frozen for up to 180 days. Beginning on 15 May 2024, 50 *Ac. saccharum* seeds were added to each plate (Tufts: 400 seeds/treatment, URI: 500 seeds/treatment). All plates were immediately returned to a refrigerator for cold stratification at 3–4°C and removed every 2–3 days for seed germination checks. A new set of treatment and control petri plates was prepared every five (Tufts) or seven (URI) days, and the seeds were carefully transferred into the new plates. This ensured consistent seed exposure to mucus in case of absorption or evaporation. Germination checks and the replacement of cue/control plates continued at Tufts until 12 weeks, once a week went by without a new germinant. Germination checks continued at URI for 18 weeks; a pilot experiment found that *Ac. saccharum* germination typically does not occur past 17 weeks of cold stratification.

#### Seed vulnerability bioassay

At URI, three weeks after treatment initiation and before any seed germination, we conducted bioassays to assess whether treatment and control seeds differed in their vulnerability to slug herbivory. Ten of the 50 seeds in each plate were haphazardly selected and removed for use in the bioassay.

Each bioassay was conducted in a 90 mm petri plate. Five treatment seeds, each from a different treatment plate, were selected and individually weighed; the same was done with five control seeds. In even-numbered bioassay plates, each of the treatment seeds was then dotted with red nail polish, and each of the control seeds was dotted with blue nail polish (Sally Hansen Insta-Dry in either ‘That’s A-Blazing’ or ‘Up in the Clouds’ respectively). In odd-numbered bioassay plates, treatment seeds were dotted with blue and control seeds with red; we did this to ensure that ‘treatment/control’ responses were not conflated with nail polish colour. A total of 20 bioassay plates were prepared, each with five treatment and five control seeds. We then added two pre-weighed mature *Ar. subfuscus* (∼1.0 g each) that had been starved for the previous 24 h to each plate. After 24 h, we reweighed each slug and each seed and recorded the presence or absence of rasping/seed coat damage.

#### Radicle susceptibility bioassay

Once at least one seed had germinated in each of the treatment and control petri plates, we removed one seed from each plate for a radicle bioassay. The treatment and control seeds in each bioassay were matched by germination date whenever possible (∼80% of replicates). Different colours of nail polish were used to distinguish the treatment and control seeds; the colour scheme was reversed in half of the replicates to ensure that treatment was not conflated with polish colour. After weighing each seed and measuring the length of each radicle, we then added one pre-weighed mature *Ar. subfuscus* that had been starved for the prior 24 h to each plate. Each bioassay lasted six hours; at URI only, plates were checked hourly, and the bioassay stopped early if at least one radicle had been consumed. We then reweighed each slug and each seed and remeasured the length of each radicle. There were 57 replicate bioassays in total (Tufts: 40, URI: 17).

### Seedling experiments

#### Planting and seedling care

Because of low germination rates at URI (51 of 1000 seeds; 5.1%), seedling experiments only occurred at Tufts. On 5 July 2024, 67 days after the beginning of cold stratification, 240 germinated seeds (120 from each treatment) were planted in 25-cm container pots (Steuwe & Sons, Tangent, OR) in a 2:2:1 ratio of unfertilized topsoil, peat moss, and sand in the Tufts University greenhouse. All 240 seeds germinated between days 46–65 of stratification. Once planted, each of the 240 seeds was assigned to one of three seedling treatments: control, slug mucus, or methyl jasmonate (described below). The two seed treatments, crossed with three seedling treatments, generated six seed + seedling treatment combinations (40 replicates/treatment). The pots were stored in 20 racks of 12 individuals; each rack contained two replicates of each treatment combination. Pots were watered three times per week with 60 ml of water, and 20 ml of Miracle-Gro NPK fertilizer was applied weekly. A total of 91 seedlings survived to the end of the experiment, and all treatments were represented with adequate sample size for the remaining assays.

#### Seedling cue exposure

To generate the seedling mucus cue treatment, 50 mm petri dishes were filled with 4.5 g of the 7:2 water: soil mixture described above. A mature *Ar. subfuscus* slug (0.75 ± 0.25 g) was added to a third of the dishes (the remaining dishes were slug-free controls) before they were held in a dark cabinet. After 24 h the slugs were removed and the treatment and control dishes frozen at −20°C until the day before cue application.

Seedling cue treatments began on 16 July 2024, once enough seedlings had emerged. Each plant first had 4.5 g of soil added to their pot and received 40 ml of water. ‘Mucus’ seedlings received mucus-covered soil, while ‘control’ and ‘methyl jasmonate’ seedlings received mucus-free soil. Immediately following soil addition, plants in the methyl jasmonate treatment were moved to a separate room of the greenhouse and sprayed with a deionized water solution containing 5 mM methyl jasmonate in 0.125% Triton X-100. In previous experiments with sugar maple seedlings, this concentration of methyl jasmonate has not strongly inhibited growth or led to leaf yellowing (unpublished data). Plants in the control and mucus treatments were sprayed with deionized water containing 0.125% Triton X-100. Plants were sprayed until the leaves were covered with droplets but before drip-off. Once water was no longer visible on the leaves, plants in the methyl jasmonate treatment were returned to their original spots. Cues were applied every 3 days for a total of five applications.

#### Harvest

One day after the final seedling cue application on 29 July 2024, shoot height (cotyledon scar to the shoot apex), leaf number, and leaf length and width for the first leaf pair were recorded for each plant. The plants were randomly sorted into harvest groups. Half were harvested on 30 July 2024 and half on 1 August 2024 by removing the first leaf pair from each plant (cutting where the petiole met the leaf blade). Leaves were kept in a cooler until use; one leaf from each pair was used for herbivore feeding assays and the other for chemical analysis.

#### Foliar susceptibility bioassay

We ran four non-choice feeding assays, two using *Ar. subfuscus* slugs and two using third instar *Lymantria dispar* caterpillars. Previous work in the group suggested that it might be difficult to get slugs to eat foliar samples in petri dishes, so feeding trials with the more reliable caterpillar species were also performed. Two trials were conducted on each harvest day, one with slugs and one with caterpillars. Mature slugs were selected for the feeding trial and were starved for 24 h; caterpillars were starved for 16 h. To prep foliar tissue for the bioassay, a 5 mm diameter cork borer was used to punch out multiple discs from each leaf. In general, two discs were used for a slug trial and two for the caterpillar trial; discs from leaves only large enough to generate two discs were only used in the caterpillar trial. The area of each leaf disc was confirmed using LeafByte (Getman-Pickering et al., 2020) before they were stored in petri dishes with moistened filter paper. Each petri dish contained two discs from the same leaf. In addition to the petri dishes with sample leaves, each trial had four petri dishes with two 5 mm leaf discs of organic iceberg lettuce (*Lactuca sativa* L. var capitata) as a control to ensure that herbivores would eat under the bioassay conditions.

Once dishes with leaf discs were completely prepped, each herbivore was weighed and then placed into a petri dish. Dishes were checked regularly to assess consumption. Caterpillar feeding trials were run for 4 h, and slug feeding trials were run overnight, for 22 h, as the caterpillars ate quicker. Leaf discs were removed at the end of each assay and the remaining area of each leaf disc measured using LeafByte to calculate herbivore consumption of treatment and control leaves.

#### Chemical analysis

Leaves assigned to chemical analysis were removed from the cooler and dried in a drying oven at 60°C for 48 h. The dry tissue was stored at −20°C before being ground with a Retsch MM400 ball mill grinder (Haan, Germany). Sub-samples of leaf tissue were extracted in methanol. Tissue extracts were then run through a Folin-Ciocalteau protocol in triplicate and run on a spectrophotometer at 765 nm to determine total phenolic content (Singleton and Rossi 1965).

### Statistical analysis

All data were analysed using RStudio 4.5.2 (R Core Team 2025). The tidyverse and rstatix packages were used for data management, and the tidyverse package was used for figure generation (Wickham et al. 2019, Kassambara 2023). When appropriate, the assumption of normality was tested using the shapiro.test function and the assumption of equal variance was tested using the leveneTest function in the car package (Fox and Weisberg 2019). Germination success for mucus and control-treated seeds was compared using a χ^2^ contingency table analysis using the chisq.test for both Tufts and URI assays. Seed treatment effects on time to radicle emergence in germinating seeds were evaluated using a two-tailed Wilcoxon rank-sum test with the wilcox.test function for both Tufts and URI assays. For the seed bioassay, seed treatment effects were analysed by taking seed weight changes for control and mucus-treated seeds, subtracting expected mass change due to evaporation, and comparing the mean weight changes using a two-sample, two-tailed Welch’s *t*-test with the t.test function. For the radicle bioassays, which were choice assays, the length of radicle that was consumed was compared between the mucus and control treatment using a paired *t*-test, also with the t.test function for both the Tufts and URI experiments.

All seedling experiments were performed at Tufts alone. Seedling emergence in the greenhouse growing trial was also analysed using a χ^2^ contingency table analysis. Groups were seed treatment, mucus or control, and whether the seedling emerged or not from the soil. The effects of seed cues and seedling cues (mucus, methyl jasmonate, control) on shoot height (cm) were evaluated with a two-way ANOVA test using the aov and Anova functions. Post-hoc comparisons were made using a Tukey’s honest significant difference test with the TukeyHSD function. The effects of seed and seedling cues on foliar phenolic concentration measured in mg of phenolic compounds per mg of dried foliar tissue were also assessed using a two-way ANOVA test, on log-transformed data. Post-hoc comparisons were performed using a Tukey’s honest significant difference test. The effects of seedling cues on foliar resistance to herbivory, measured as percent consumption of leaf tissue by herbivores, were analysed using a Kruskal-Wallis test with Dunn’s test for post-hoc comparisons with the kruskal.test function. Finally, a Spearman’s correlation test was used to directly analyse the relationship between total phenolic concentration and foliar consumption percentage using the cor.test function.

## Results

### Germination assays

**Figure 1:**
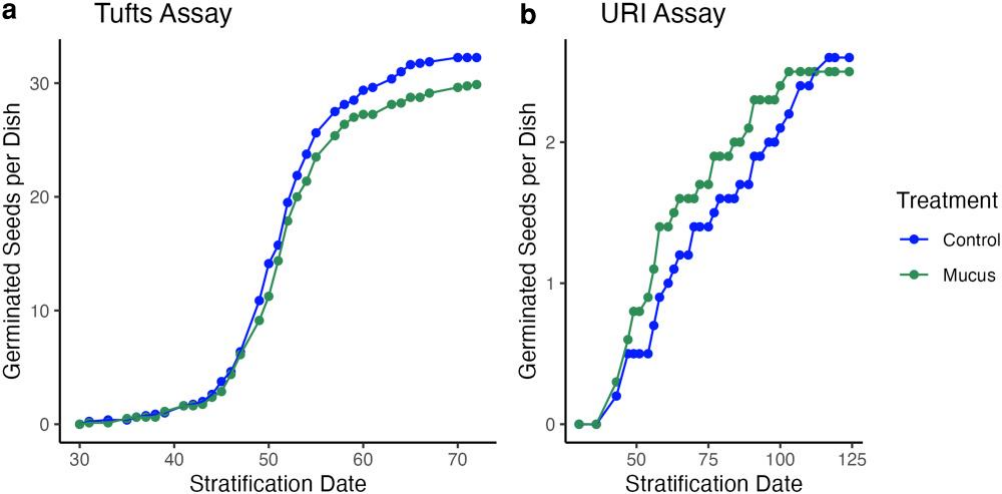
Average number of germinated seeds per petri dish for each treatment in Tufts (a) and URI (b) germination assays.

Parallel germination trials at Tufts and URI showed that slug mucus cues had no effect on sugar maple germination (Fig. 1). The Tufts germination trials had an overall germination rate of 62.1% and the URI trials had a germination rate of 5.1%. Both germination assays found that mucus exposure did not affect germination speed (Tufts: Wilcoxon rank sum test, *W* = 30075, *n* = 497, *P* = 0.64; URI: Wilcoxon rank sum test, *W* = 408, *n* =51, *P* = 0.12) or the odds of germination (Tufts: chi-squared contingency table test, χ^2^_1df_ = 1.72, *P* = 0.19; URI: chi-squared contingency table test, χ^2^_1df_ = 0, *P* = 1.00).

### Seed bioassay

**Figure 2:**
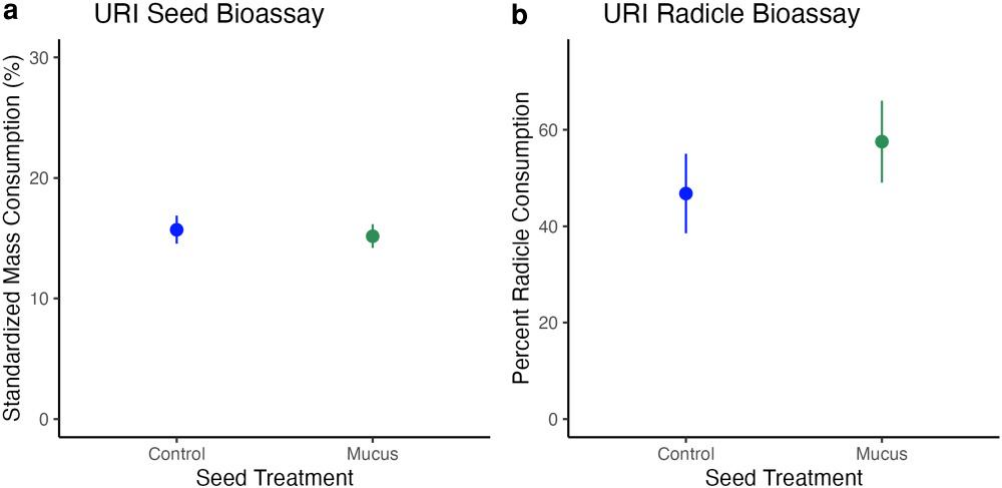
(a) Percent seed mass lost (mean ± SE) during seed bioassay treatment, standardized for average mass lost due to drying. (b) Percent radicle length (mean ± SE) consumed during radicle bioassay.

*Ar. subfuscus* seed consumption was unaffected by prior exposure to *Ar. subfuscus* mucus (Fig. 2a, Welch two sample two-tailed *t*-test, *t* = 0.35, df = 37.05, *P* = 0.73). Seed bioassays were only performed at URI.

### Radicle bioassay

In both radicle bioassays, seed exposure to mucus did not affect *Ar. subfuscus* foraging preference. Slugs consumed the same amount of radicle length on mucus treated seeds and control seeds (Tufts: two-tailed paired *t*-test, *t* = 1.12, df = 26, *P* = 0.273; URI: Fig. 2b, two-tailed paired *t*-test, *t* = 0.11, df = 10, *P* = 0.916). In both experiments, approximately two-thirds of the slugs in the bioassays consumed radicle tissue (Tufts: 67.5%, URI: 64.7%).

### Seedling performance

**Figure 3:**
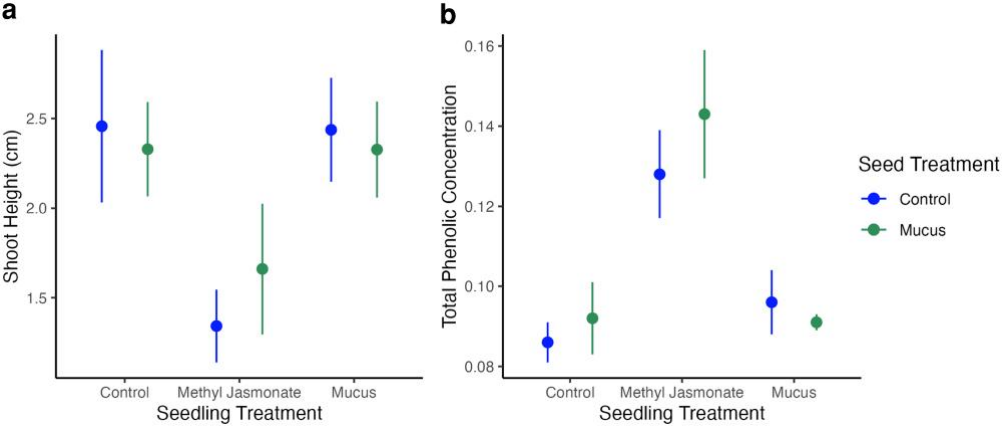
(a) Shoot height comparison (mean ± se) of seed and seedling cue treatments after Tufts greenhouse growth experiment. (b) Comparison of total phenolic compound concentration (mean ± se) between different seed and seedling cue treatments. Total phenolic concentration is measured in mg of gallic acid equivalent per g of foliar tissue.

Slug mucus did not affect seedling emergence in the greenhouse growth experiment (Chi-squared contingency table test, χ^2^_1df_ = 3.47, *P* = 0.06). Seedling shoot height was significantly affected by herbivory cues applied at the seedling stage but not by those applied at the seed stage (Fig. 3a, Two-way ANOVA; seed cues: F_1,84_ < 0.01, *P* = 0.950; seedling cues: F_2,84_ = 6.29, *P* = 0.003; interaction: F_2,84_ = 0.32, *P* = 0.728). Specifically, seedlings treated with methyl jasmonate (MeJa) were significantly shorter than control seedlings (*P* = 0.009) and mucus-treated seedlings (*P* = 0.007).

### Foliar chemistry

Total phenolic concentration was significantly affected by seedling cues, but there was no effect of seed cues or their interaction (Fig. 3b, Two-way ANOVA on log-transformed data, seed cues: F_1,54_ = 0.40, *P* = 0.531, seedling cues: F_2,54_ = 12.45, *P* < 0.001, interaction: F_2,54_ = 0.22, *P* = 0.801). Phenolic concentration was significantly higher in MeJa-treated seedlings than in control (*P* < 0.001) or mucus-treated seedlings (*P* < 0.001).

### Foliar susceptibility bioassays

**Figure 4:**
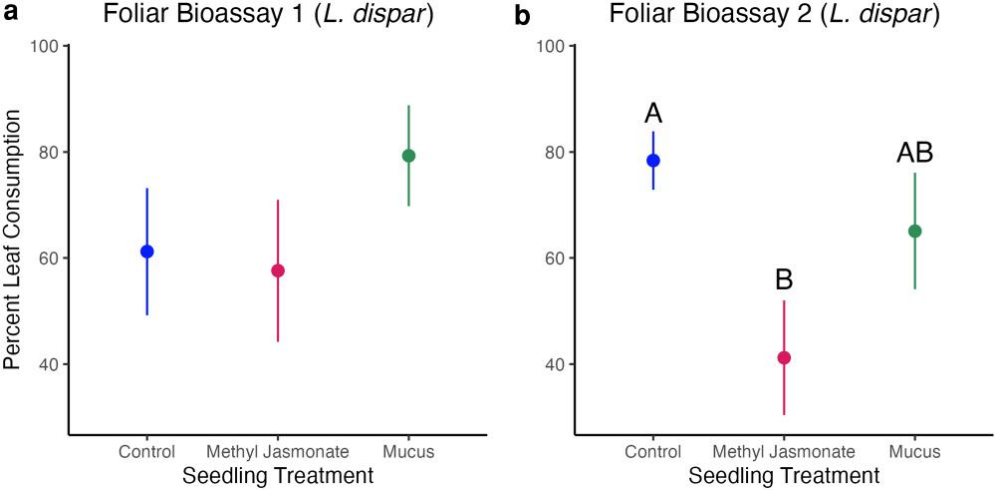
Comparison of percent consumption of leaf discs (mean ± SE) by seedling treatments in *L. dispar* (spongy moth caterpillar) feeding trials.

Slugs in the *Ar. subfuscus* foliar bioassays did not consume enough sugar maple tissue to test for treatment effects. The two trials with *L. dispar* (spongy moth) caterpillars showed different responses. In the first caterpillar trial (Fig. 4a), there was no significant effect of seedling cues (Kruskal–Wallis, χ^2^ = 3.51, df = 2, *P* = 0.173). However, in the second caterpillar trial (Fig. 4b) there was a significant effect of seedling cues on consumption (Kruskal–Wallis, χ^2^ = 6.64, df = 2, *P* = 0.036). Specifically, with the results of the post-hoc Dunn’s test, methyl jasmonate-treated seedlings were eaten significantly less than control seedlings (*P* = 0.037). However, methyl jasmonate-treated seedlings were not eaten at a significantly different rate as mucus-treated seedlings (*P* = 0.116), and mucus-treated seedlings were consumed at a similar rate to control seedlings (*P* = 0.547). We note that methyl jasmonate-treated foliar tissue was fed upon the least in both trials. There was also a marginally significant negative correlation between leaf consumption and total phenolic concentration per seedling (Spearman’s correlation test, S = 44207, ρ = −0.228, *P* = 0.079; data not shown).

## Discussion

Pre-attack cues provide organisms with valuable information about risk that allows them to respond to threats before damage has occurred. While mature plants are generally tolerant of low to moderate levels of herbivory, seeds and seedlings are more vulnerable because of their small size and limited energetic reserves. Existing research has found that both seeds (Zolovs et al. 2022, Pellegrini et al. 2024) and seedlings (Orrock 2013, Falk et al. 2014, Orrock et al. 2018, Overstrum et al. 2026) of herbaceous plants respond to the threat of attack from an herbivore by increasing resource allocation to defence. However, there is a gap in our understanding of the use of pre-attack cues by woody seeds and seedlings. While the susceptibility of woody seeds and seedlings to herbivory is well known (Nystrand and Granstrom 2000, Albrectsen et al. 2004, Lorca et al. 2019), and there exists plentiful research about the effect of herbivory on induced plant defences (Karban and Baldwin 1997, Karban et al. 1999, Calf et al. 2020), we are unaware of any other experiments addressing whether woody seeds and seedlings, like their herbaceous counterparts, respond to pre-attack herbivore cues.

Our parallel experiments found broadly similar results: prior exposure of *Ac. saccharum* seed and seedlings to *Ar. subfuscus* mucus did not affect seed germination or foliar phenolic concentration, the preference of *Ar. subfuscus* slugs for *Ac. saccharum* seeds or radicles, or *L. dispar* preference for *Ac. saccharum* leaves. These results are counter to what previous research on herbaceous plants has found (Orrock 2013, Orrock et al. 2018, Zolovs et al. 2022, Pellegrini et al. 2024), which is that herbaceous seeds and seedlings respond to the threat of herbivory communicated through kairomone exposure. The fact that two well-replicated experiments yielded similar results suggests a genuine lack of response in *Ac. saccharum* seeds and seedlings to pre-attack *Ar. subfuscus* cues. Though several of the assays were performed with seeds with a significantly lower germination rate, the consistency of their results with other assays lends credence to the finding of a lack of effect of mucus exposure. While null results that do not support expectations are less likely to be published (Rosenthal 1979), we are confident that our high replication and two experiments conducted under similar conditions in two separate locations provide strong support for the validity of our null result. We use retrospective power analyses to assess the effectiveness of our study design in the supplementary information document.

We have three potential alternative explanations for our results. First, *Ac. saccharum* seeds might have habituated to the slug mucus cue and thus diminished their response. Previous studies (Zolovs et al. 2022, Pellegrini et al. 2024) exposed seeds to mucus cues for much shorter periods than the 7–18 week exposure in our study. Chronic exposure to a risk cue without attack may diminish the potential threat that the *Ar. subfuscus* mucus represents. Previous research has found that mosquito larvae prey habituate to damselfly and dragonfly kairomones (Roberts 2014), and continuous exposure to bat predator ultrasound caused moths (Gillam et al. 2011) and crickets (Engel and Hoy 1999) to habituate to the sound. We are unaware, however, of any research exploring seed and seedling habituation to herbivore kairomones.

Second, the timing and duration of plant responses to herbivory are highly variable across scales (Underwood 1999). Individual plants can vary greatly in nutritive traits (Wetzel et al. 2016), and plants of the same species growing in the same area can vary greatly in physical defence (Wetzel and Meek 2019) and susceptibility to herbivore damage (Wetzel et al. 2023). While our mean responses of seed/seedling exposure to herbivore kairomones did not differ significantly between treatment groups, our study did not look at individual variability across space and time; as Wetzel et al. (2023) note, variability-explicit research remains in early stages in the study of herbivory.

The lack of response may also reflect the fact that *Ac. saccharum* did not co-evolve with *Ar. subfuscus*. *Ac. saccharum* is native to the eastern United States and Canada (Burns et al. 1990, Payette et al. 2018), while *Ar. subfuscus* is native to Europe and was first recorded in the United States during the 19th century (Chichester and Getz 1969). Because *Ac. saccharum* can reach 300 to 400 years of age (Burns et al. 1990), there may not have been enough co-occurring generations of the two species for *Ac. saccharum* to evolve the ability to detect *Ar. subfuscus* mucus. It would be fascinating to see whether replicating this experiment with species that share their native ranges (and thus presumably a coevolutionary history) would yield similar results. It may be that using a woody plant species, such as *Acer platanoides* in Europe, that has co-evolved with *Ar. subfuscus*, would result in the woody plant species being better able to detect the herbivore’s kairomones.

Our experimental design, although producing interesting results, had some limitations. For example, the laboratory and greenhouse conditions under which our series of experiments were performed is not representative of the conditions under which sugar maples stratify, emerge, and grow. However, our protocols mimicked those of Pellegrini et al. (2024), which prompted a response to mollusc kairomones in herbaceous plants. Further validation of responses in a field environment are necessary to fully understand how plants utilize slug kairomones, but we can still assess whether woody species can detect kairomones with these procedures. Another limitation is that different developmental stages could respond differently to kairomones, so it is possible that we might miss a window in which sugar maple seedlings are more likely to respond to mucus cues. However, given that the tree seed and seedling stages are those with the highest levels of mortality (Harcombe 1987), these early stages would be the best candidates for a response to slug kairomones to occur, given the additional selection pressure. An additional limitation of our study design is that it included limited defence measurements, only focusing on the conglomeration of all phenolic compounds, instead of looking at multiple types of chemical and physical defences. While it is ideal to measure these many forms of plant defences, total phenolics are considered to be a key element of foliar defence in trees (Barber and Fahey 2015). We found evidence suggesting as much in this study, as total phenolic concentration had a marginal negative correlation with foliar damage from caterpillars.

The high variability in germination success between the experiments at Tufts and URI was surprising. Previous work in the group has demonstrated that the typical germination rates in experiments sit between 50% and 70% (unpublished data). The trial at Tufts fell in this typical range with 62.1% germination, which shows kairomones have no effect on germination in a typical germination year, and the trial at URI shows that even when germination falls below expected, that result still holds. One final potential limitation of our study was that slugs were not starved prior to mucus collection. A plausible reason for this concern is that it has been shown that when a slug is fed a tissue from a conspecific plant, their mucus has a more pronounced effect as a kairomone than mucus from a slug fed on other plant tissue (Overstrum et al. 2026). However, the slugs in the study were fed lettuce and carrots, which are different plant species. Plus, the consistent access to food also made it so that slug faeces and frass were included with the collection of the mucus, similar to a natural setting, in case those excretions were relevant in plant responses to kairomones.

In summary, kairomones are typically studied in the context of predator-prey relationships (Clinchy et al. 2013, Karban et al. 2016), and the existing body of research on plant responses to herbivore kairomones is much smaller (but see: Orrock 2013, Orrock et al. 2018, Pellegrini et al. 2024). Our research demonstrates that *Ac. saccharum* seeds and seedlings do not react to the presence of the herbivore *Ar. subfuscus* kairomones. However, invasive members of the *Arion* genus are projected to increase their invaded range in the United States (Zemanova et al. 2018), so they will exert further herbivory pressure on woody seeds and seedlings. Therefore, it is important that we continue to study how woody seedlings defend against mollusc herbivory generally. In addition, our study only focused on the impacts of herbivore kairomones on a singular woody plant species; woody plants vary substantially, and further research is needed to determine the response of other woody plant species to herbivore kairomones.

## Supplementary Material

plag013_Supplementary_Data

## Data Availability

Raw data are available online at 10.6084/m9.figshare.29610335 and R code is available at 10.6084/m9.figshare.31255144.
