# Supplementary material for: Woody seeds and seedlings are unresponsive to herbivore kairomones
Source: AoB Plants. 2026 Feb 26;18(2):plag013. doi: 10.1093/aobpla/plag013 (PMC12980128; doi:10.1093/aobpla/plag013)
Supplement: plag013_Supplementary_Data [file plag013_supplementary_data.pdf]

Supporting Information

Slug Feeding Trials

Methods for these feeding trials are found in the main text of the manuscript. It was suggested to re-analyze the data with tissue marked as undamaged or damaged. Given that this new data is binary, a two-way ANOVA test is no longer, as the test requires a normally distributed response variable. Instead, we ran a Kruskal-Wallis test, which has no such assumption. We found that in both trials, there was no significant effect of any treatment on the likelihood of damage to foliar tissue by slugs (Trial 1: Kruskal-Wallis Test, Fig S1, $\chi^2 = 6.76$, df = 5, p = 0.239; Trial 2: Kruskal-Wallis Test, Fig S2, $\chi^2 = 6.21$, df = 5, p = 0.286).

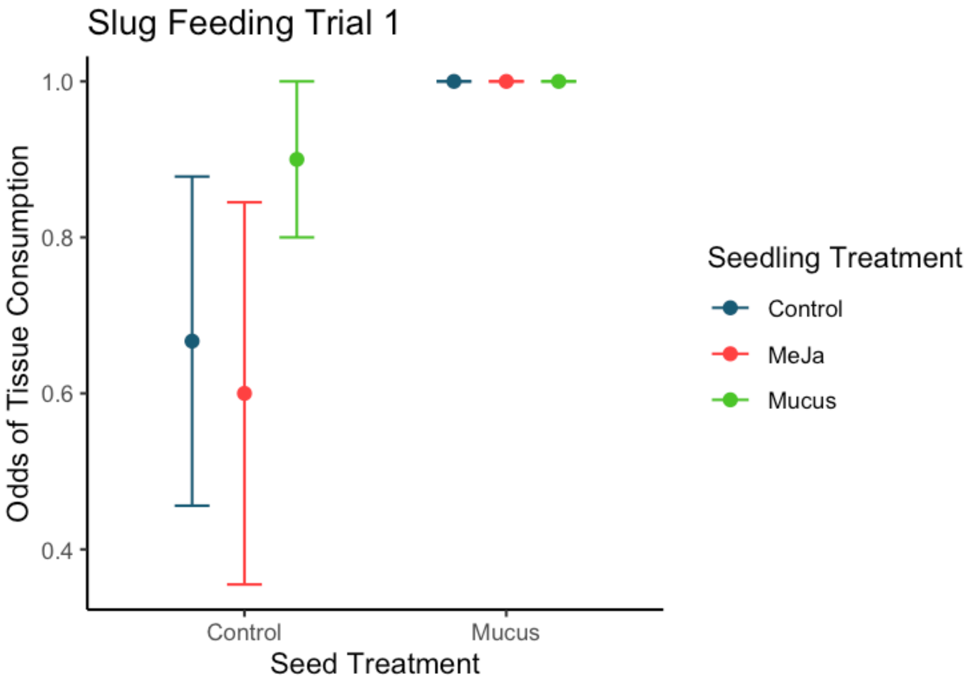

Figure S1. Effects of herbivory cues applied to seeds and seedlings on odds of consumption by *Arion subfuscus* slugs in the 30 July 2024 feeding trial. There were no significant effects.

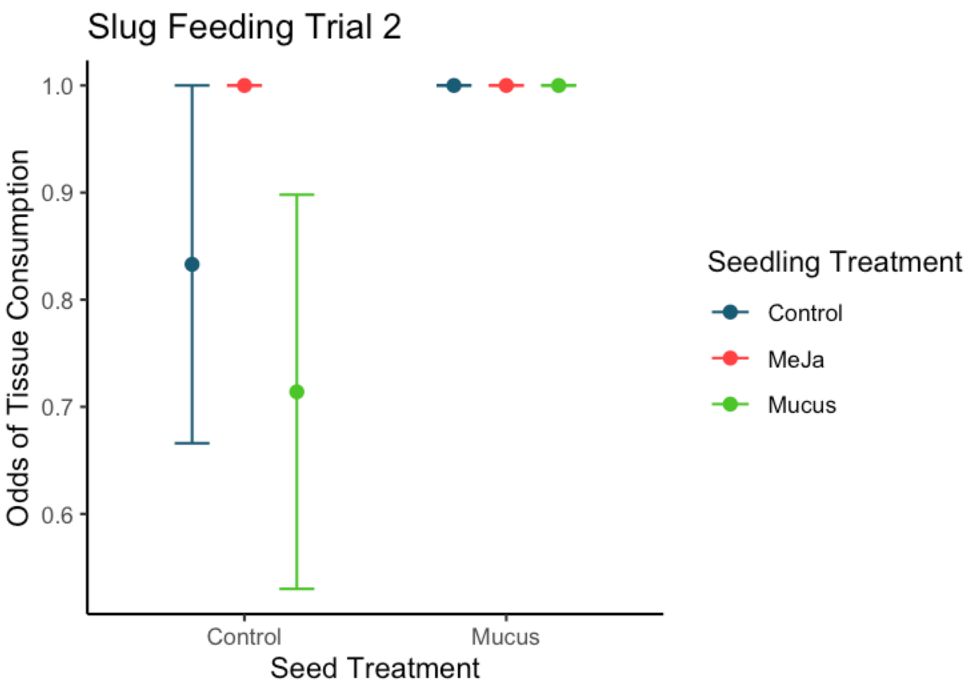

Figure S2. Effects of herbivory cues applied to seeds and seedlings on odds of consumption by *Arion subfuscus* slugs in the 1 August 2024 feeding trial. There were no significant effects.

Power Analyses

For all power analyses, we calculated the necessary effect size to obtain a significant p-value at the level of 0.05 with power of 0.8, which is considered the standard for a high-power statistical test (Cohen 1992).

Germination Assays

To analyze the power of the germination assays, we used the pwr.chisq.test function in the pwr package for chi-squared goodness of fit tests and the shiehpow function in the wmwpow package for the Wilcoxon rank-sum tests. For the tests of germination success, we found that both the trials at Institution X (n=800) and Institution Y (n = 1000) only required negligible effect sizes to produce a significant result (0.099 and 0.089, respectively). For analyses of germination speed, the assay at Institution X (n1 = 258, n2 = 239) only required a negligent effect size $p < 0.001$ to achieve sufficient power. At Institution Y, which had markedly lower germination rates (n1 = 25, n2 = 26), the necessary effect size to achieve significance of sufficient power was 0.280, which is higher, though still considered a low effect size.

Seed and Radicle Bioassays

All seed and radicle bioassays power analyses were performed with the pwr.t.test function in the pwr package. In the seed bioassay at Institution Y (n1 = n2 = 20), the necessary effect size to achieve the sufficient level of power is 0.909, which is a large effect size. For the radicle bioassays at Institution X (n1 = n2 = 27), the necessary effect size is 0.560, which a medium

effect size. At Institution Y (n1 = n2 = 11), the decreased sample size leads a large necessary effect size of 0.938.

Seedling Trait Assays

All seedling assays took place at Institution X alone. The seedling emergence power analysis also required the pwr.chisq.test function from the pwr package. For seedling emergence (n = 240) the necessary effect size to achieve the desired power was low, at 0.181. The power analyses for shoot height and foliar chemistry both required the ss.2way function from the pwr2 package to analyze the two-way ANOVA tests. Both statistical tests had two factors: seed treatments with 2 levels and seedling treatments with 3 levels. For shoot height, which had an average group size of 14, the necessary effect size to achieve acceptable power was a low-medium level 0.34. The foliar chemistry assay had a slightly lower average group size of 10, and therefore had a slightly necessary effect size, a medium level 0.42. For both shoot height and foliar chemistry, methyl jasmonate treatment created a significant difference, but there was no significant effect of slug mucus treatment.

Resistance Assays

The power analyses for foliar resistance to caterpillar herbivory bioassays were performed with the ss.1way function in the pwr2 package. The two trials both consisted of the three levels of a single factor, seedling treatments, and both had an average of 13 individuals in each group. They also had similar overall variation and group differences, which led to the same necessary effect size for both assays, of 0.52. It is a medium effect size, but these analyses are meant for one-way ANOVAs, but the data were analyzed using Kruskal-Wallis tests, so the necessary effect size would likely be larger, due to the decreased power of the Kruskal-Wallis test when compared with ANOVA.

Conclusion

With the exception of the seed and radicle bioassays at Institution Y, all the power analyses revealed that the necessary effect sizes were either very low or in a few cases medium-sized. Therefore, the design of our study provided ample opportunity to detect an effect of slug mucus as a putative kairomone across several early life stages in sugar maples. This provides confidence in our consistent non-significant findings, which indicates that sugar maples are unable to detect slug mucus as a predictor of future attack and respond with growth and defense responses.

References

Cohen, J. (1992). Quantitative methods in psychology: A power primer. *Psychol. Bull.*, *112*, 1155-1159
